# Towards a deeper understanding of structural biomass recalcitrance using phase-contrast tomography

**DOI:** 10.1186/s13068-015-0229-8

**Published:** 2015-03-10

**Authors:** Augusta Isaac, Vinicius Barboza, Federico Ivan Sket, José Roberto M D’Almeida, Luciano Andrey Montoro, André Hilger, Ingo Manke

**Affiliations:** Department of Metallurgical and Materials Engineering, Universidade Federal de Minas Gerais - UFMG, Avenida Presidente Antônio Carlos, 6627, 31270-901 Belo Horizonte, MG Brazil; Institute of Computing, Universidade Estadual de Campinas - UNICAMP, Avenida Albert Einstein, Cidade Universitária Zeferino Vaz - Barão Geraldo, 13081-970 Campinas, SP Brazil; Instituto Madrileño de Estudios Avanzados - IMDEA, E.T.S. de Ingeniería de Caminos C/ Profesor Aranguren, 28040 Madrid, Spain; Materials Engineering Department, Pontifícia Universidade Católica do Rio de Janeiro, Rua Marques de São Vicente, 225, Gávea, 22451-900 Rio de Janeiro, RJ Brazil; Department of Chemistry, Universidade Federal de Minas Gerais - UFMG, Avenida Presidente Antônio Carlos, 6627, 31270-901 Belo Horizonte, MG Brazil; Institute of Applied Materials, Helmholtz-Zentrum Berlin, Hahn-Meitner-Platz 1, 14109 Berlin, Germany

**Keywords:** Biomass, Surface area, Recalcitrance, Phase-contrast tomography, Synchrotron radiation

## Abstract

**Background:**

The development of technological routes to convert lignocellulosic biomass to liquid fuels requires an in-depth understanding of the cell wall architecture of substrates. Essential pretreatment processes are conducted to reduce biomass recalcitrance and usually increase the reactive surface area. Quantitative three-dimensional information about both bulk and surface structural features of substrates needs to be obtained to expand our knowledge of substrates. In this work, phase-contrast tomography (PCT) was used to gather information about the structure of a model lignocellulosic biomass (piassava fibers).

**Results:**

The three-dimensional cellular organization of piassava fibers was characterized by PCT using synchrotron radiation. This technique enabled important physical features that describe the substrate piassava fibers to be visualized and quantified. The external surface area of a fiber and internal surface area of the pores in a fiber could be determined separately. More than 96% of the overall surface area available to enzymes was in the bulk substrate. The pore surface area and length exhibited a positive linear relationship, where the slope of this relationship depended on the plant tissue.

**Conclusions:**

We demonstrated that PCT is a powerful tool for the three-dimensional characterization of the cell wall features related to biomass recalcitrance. Original and relevant quantitative information about the structural features of the analyzed material were obtained. The data obtained by PCT can be used to improve processing routes to efficiently convert biomass feedstock into sugars.

## Background

Biomass is the only domestic, sustainable, and renewable primary energy fuel source in the near term. Alternative energy initiatives have now rekindled enormous interest in the development of new and cost-effective processes for converting plant-derived biomass to liquid fuels. This combined effort is global, serious, and hopefully long lasting. A major component of the current focus on alternative energy is associated with environmental issues, particularly with the huge effect of the usage of fossil fuels on the carbon cycle and greenhouse gas emissions. Although initially debates raged, the promise and appropriateness of biofuels are now clearly apparent.

A biorefinery is envisioned to contain four major sections: feedstock harvest and storage, thermochemical pretreatment, enzymatic hydrolysis, and sugar fermentation to ethanol or other fuels. Existing biomass conversion schemes typically rely on a combination of chemical and enzymatic treatments. A pretreatment step is usually conducted to reduce recalcitrance by depolymerizing and solubilizing hemicellulose and lignin. In addition, pretreatment typically breaks down the rigidity of biomass and decreases the physical barriers to mass transport [1]. However, the chemical and enzymatic conversion processes developed during the past 80 years are still expensive. The high costs of these processes are, in part, because of the limited knowledge of the bulk structure of the substrate itself and the influence of pretreatment processes on the accessibility of the β-glycosidic bonds in cellulose to cellulase enzymes.

Cellulase enzymes must bind to the surface of substrate particles before hydrolysis of insoluble cellulose can occur. The three-dimensional structure of such particles (including their microstructure) and the size and shape of cellulase enzyme(s) are the main limiting factors in the enzymatic hydrolysis of lignocellulosic biomass [2,3]. Cellulosic particles are typically heterogeneous porous substrates, and their available surface area can generally be divided into external and internal surface areas. The external surface area of cellulosic-rich materials is largely determined by the overall dimensions of individual fibers [4,5]. The internal surface area of porous cellulose particles consists of internal pores, fissures, and microcracks, which typically arise from discontinuities in the molecular packing of cellulose generated during the formation of the solid substrate or surface openings/internal slits, voids, or spaces formed by the removal of noncellulosic cell wall components during pretreatment [4,5]. In general, the internal surface area of cellulose is 1 to 2 orders of magnitude higher than its external surface area [2,4].

The external surface area of a material is closely related to the particle shape and size and has been estimated by laser diffraction and electron microscopy techniques [6-11]. Conventional scanning electron microscopy (SEM) does not allow observation of the bulk of materials because it provides information based only on two-dimensional topographical images. In addition, microscopy techniques involve extensive and time-intensive sample preparation that can potentially change the native structure of the plant cell wall [12].

The gross surface area of a material is generally measured by its sorption of nitrogen, argon, or water vapor [2]. The most widely used procedure to determine specific surface area is Brunauer-Emmett-Teller (BET) analysis of nitrogen adsorption measurements. However, the gross accessibility of cellulose to enzymes is only a relatively small fraction of the total surface area measured by these techniques [2,4]. Additionally, because of variations in experimental conditions such as adsorption time, vacuum time and vacuum pressure [13], sample preparation [3], and sample origin and features [7,13], a wide range of gross area values for the same substrate have been reported.

Recently, conventional tomography (that is, absorption contrast) was used to explore the three-dimensional microstructure of sugarcane bagasse particles [14]. This noninvasive technique is suitable to visualize the bulk microstructure of plant tissues and their changes induced by physical and chemical processes. However, the inherent limited resolution of absorption-contrast imaging does not allow the pore volume and shape or the local cell wall thickness of lignocellulosic materials to be accurately determined [14].

In this decade, the use of X-ray phase imaging techniques including phase-contrast tomography (PCT) has attracted extensive attention from scientific and technological communities [15]. The interaction cross section of X-ray phase shift is much larger than that of absorption, so extremely high sensitivity can be achieved for weakly absorbing materials like biological soft tissues. PCT is also attractive because even tiny structures produce phase contrast clearer than absorption contrast, leading to higher spatial resolution [15]. As a result, PCT has emerged as a powerful technique to investigate such materials at the submicrometer scale and three-dimensional perspective with essentially no sample preparation or perturbation. In this work, the ability of PCT to image biomass is presented and discussed. The benefits of combining PCT with image processing techniques to provide quantitative information about cellular structure are also considered.

## Results and discussion

The anatomy of plant cell walls was investigated using PCT to gain a deeper understanding of the cellular organization. In this context, piassava fibers (*Attalea funifera*) were used as a model material [16,17]. Piassava was selected instead of other renewable feedstock such as sugarcane bagasse, corn stover, and rice straw because of its stiff, resistant, and easily handled fibers that perfectly suits this characterization technique and three-dimensional analysis. The results presented in this section about cell wall structure related to biomass recalcitrance illustrate the great potential of this three-dimensional characterization technique with high spatial resolution.

### Cell wall organization

**Figure 1:**
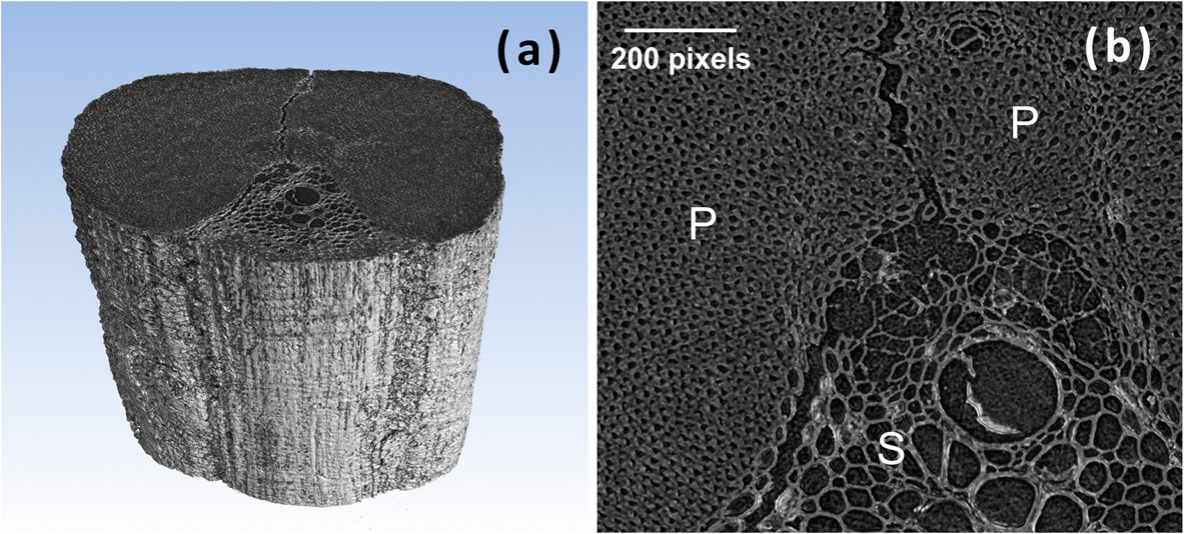
**Phase-contrast tomography (PCT) images of a piassava fiber. (a)** PCT image of a single fiber and **(b)** a magnified view of the vascular bundle and surrounding parenchyma cells. The sclerenchyma and parenchyma are indicated S and P, respectively.

The tomographic reconstruction of a piassava fiber is shown in Figure 1. The anatomical components of piassava (fiber structure and pith) are easy to identify in the three-dimensional view displayed in Figure 1a and also in the cross section of the fiber presented in Figure 1b. These images reveal not only differences in cell type and size but also the existence of empty spaces corresponding to internal lumens (pores). Each voxel (volume element of the three-dimensional data set) corresponds to dimensions of 0.438 × 0.438 × 0.438 μm.

Unlike the usual arrangement of cells in monocot stems [8,9,11], the piassava fiber has a single vascular bundle near its external surface. Similar to maize stem and sugarcane bagasse particles [8-11], the vascular bundle is surrounded by sclerenchymatous cells and embedded in the parenchyma. The sclerenchyma region (Figure 1b) exhibits irregularly thickened cell walls, containing internal lumens with diameters between 15 and 170 pixels (6.57 and 74.46 μm, respectively). Large pores are mostly caused by the merging of neighboring cells with thin walls, that is, three pixels or less (≤1.31 μm). Parenchyma cells are the most numerous cells forming the bulk of the fiber. Parenchyma cells have fairly thick walls of approximately 12 pixels (5.26 μm) and contain internal lumens ranging from about 4 to 14 pixels (1.75 and 6.13 μm, respectively) in diameter. In addition, a large crack across the particle can also be visualized (Figure 1a).

The high contrast of phase-contrast imaging means that topographic characteristics of the external surface can be observed in detail (Figure 1a). Fine surface irregularities were resolved. In a previous study [16], the surface roughness of the piassava fiber was partly attributed to the attachment of silicon-rich spinulose particles on the fiber surface.

Many of the challenges that are encountered in the detailed characterization of biomass arise because plant cell wall recalcitrance occurs on multiple length scales [12,18]. For example, vascular bundles and pith tissues are evident within the same grass stem at a scale of 10^−4^ m, different cell types may be identified at a scale of 10^−5^ m, the primary and secondary walls of individual cells may be differentiated at the scale of approximately 10^−6^ m, and the individual cellulose microfibers that make up these walls have dimensions in the order of approximately 10^−9^ m [12]. It became clear that the physical reasons for the biomass recalcitrance and its relationship to deconstruction need to be explored over this whole scale range. In particular, PCT is a promising technique for the advanced characterization of the cellular organization of important lignocellulosic biomass (for example, sugarcane bagasse and corn stover) because its spatial resolution spans many orders of magnitude and has three-dimensional capability. Here, we presented statistically relevant information about the heterogeneous structural features of biomass (Figure 1). The hierarchically structured tissues with both macroscopic and microscopic three-dimensional features were clearly visible in these PCT images [19]. Parameters that were assessed at these hierarchical levels were the size and shape of the cells and the thickness of the cell walls. In addition, the spatial distribution of vascular bundles, pith and fibers over a plant cross section was presented. But, unlike in two-dimensional imaging techniques such as SEM, these features were not investigated from surface images. The complex three-dimensional interconnectivity of the native plant structure or the results from pretreatment can be visualized through the reconstructed biomass volume, which is a more statically relevant part of the sample than that imaged by two-dimensional characterization techniques. In addition, PCT is suitable for pristine specimens and does not need expensive or time-consuming preparation methods, as are often required by electron-based characterization techniques [12].

### Quantitative information about cellular organization

Internal lumens were characterized by image processing techniques to calculate their surface area, size, and length. A similar method to quantify the size and shape distribution of particles/pores has been reported previously [20]. In addition, the local thickness tool in ImageJ was used to calculate the local cell wall thickness of the lignocellulosic structure [21,22]. Because of the physical limits of computation, the quantitative analyses were performed for a representative part of the tomogram in Figure 1a corresponding to 250 slices. Information about more than 33,000 pores was extracted.

**Figure 2:**
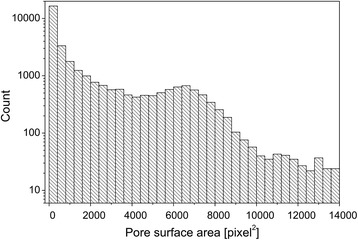
**Pore surface areas of piassava fibers.** Histogram of the surface areas of individual pores.

Figure 2 shows the number of pores as a function of the surface area of individual pores obtained from the tomogram. The histogram of pore surface area exhibits a bimodal distribution, which is a combination of an exponential decay curve with a log-normal distribution. The first peak of the bimodal distribution in Figure 2 corresponds to the pores with surface area less than 1,200 pixels squared (230.21 μm^2^), while the second peak originates from pores with surface area between 5,600 and 6,800 pixels squared (1,074.32 and 1,304.54 μm^2^, respectively). Similar bimodal distributions of biomass features have been observed at different scales. For instance, Wang *et al*. [23] and Mooney *et al*. [24] reported bimodal distributions of the particle sizes of pretreated corn stover and kraft pulp samples, respectively.

**Figure 3:**
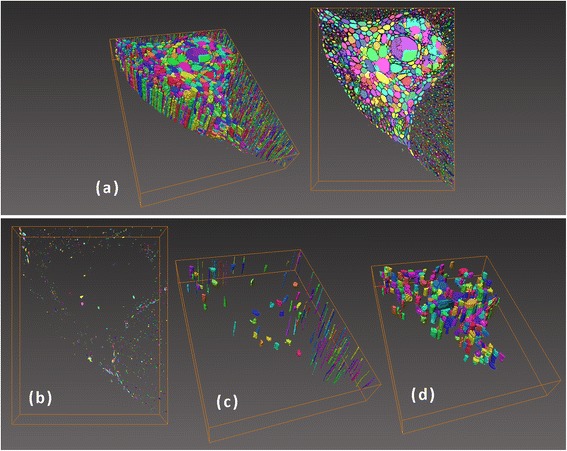
**Tomographic images of a single piassava fiber.** Images of **(a)** all of the pores in one quarter of the fiber cross section, **(b)** pores with individual surface areas of less than 1,200 pixels squared, **(c)** pores with individual surface areas between 5,600 and 6,800 pixels squared, and **(d)** pores with individual surface areas greater than 6,000 pixels squared and lengths between 40 and 120 pixels. Bounding box of dimensions of 1,400 × 1,000 × 250 pixels.

To examine the distribution of surface areas of individual pores, Figure 3 illustrates the graphic information of a quarter of the volume (that is, sectioned in area rather than height), which includes the vascular bundle, sclerenchyma, and part of the parenchyma tissue. Although quantitative analysis was performed for more than 33,000 pores, the information displayed in Figure 3 is for a representative sample. Figure 3a shows all of the pores detected in one quarter of the volume. Meanwhile, Figure 3b,c highlights the pores that contribute to the first and second peaks of the histogram of surface area distribution in Figure 2, respectively. It is clear that pores in the parenchyma tissue mainly correspond to the first peak of this histogram, while the largest pores in the parenchyma and also some pores in the sclerenchyma correspond to the second.

Because direct physical contact between an enzyme and substrate is required for hydrolysis, the amount of surface area available for such contact strongly affects the reaction rate [2,4]. In this study, the contribution of the external and internal surface areas (for example, substrate porosity, fissures, and microcracks) to the overall accessible surface area was assessed. The internal surface area was estimated by adding the surface areas of all pores detected by PCT. The external surface area of the fiber could also be computed. The overall surface area includes the fiber surface area and that of the surface of the pores visible by PCT (Table 1). These data reveal that approximately 96% of the overall surface area resolved by this technique corresponds to internal surface area inside the bulk fiber. Therefore, it seems logical that the porous structure of plants has a major influence on the diffusion of reactants into the cellulose network.

**Table 1:** **External, internal, and total surface areas of piassava fibers resolved by phase-contrast tomography**

**Type of surface area**	**Value [m** ^**2**^ **]**	**Value [m** ^**2**^ **/g]**
Internal surface area (pores)	1.4080 × 10^−5^	0.1334
External surface area (fiber)	5.0990 × 10^−7^	0.0048
Total surface area	1.4590 × 10^−5^	0.1382

Many researchers have suggested that particle length affects enzyme access to cellulose microfibrils [3,24,25]. However, several authors have shown that the area of enzyme adsorption is much larger than that of the external surface of a particle [2,26,27]. This observation is supported by the almost negligible external surface area of the particles. Isaac *et al*. [14] recently provided direct visual confirmation that pretreatment has a marked effect not only on particle length but also on internal structural features. Thus, it might be more appropriate to infer the relationship between hydrolysis rate and pore length instead of that between hydrolysis rate and particle size. Future work will concentrate on this issue.

For comparison purposes, nitrogen adsorption measurements were obtained to determine the surface area of the fibers by the BET method. This method measures both the interior and exterior surface areas where a nitrogen monolayer is adsorbed by a sample. The surface area of the fibers determined by the BET method was 0.6379 m^2^/g, which is the same order of magnitude as that obtained from PCT. However, this area was calculated based on the adsorption cross section of the nitrogen molecule. Therefore, the value obtained from nitrogen isotherms surely overestimates the surface area of a substrate accessible to enzymes [2]. Thus, the surface area probed by PCT may be more accurate when considering enzymatic kinetics.

**Figure 4:**
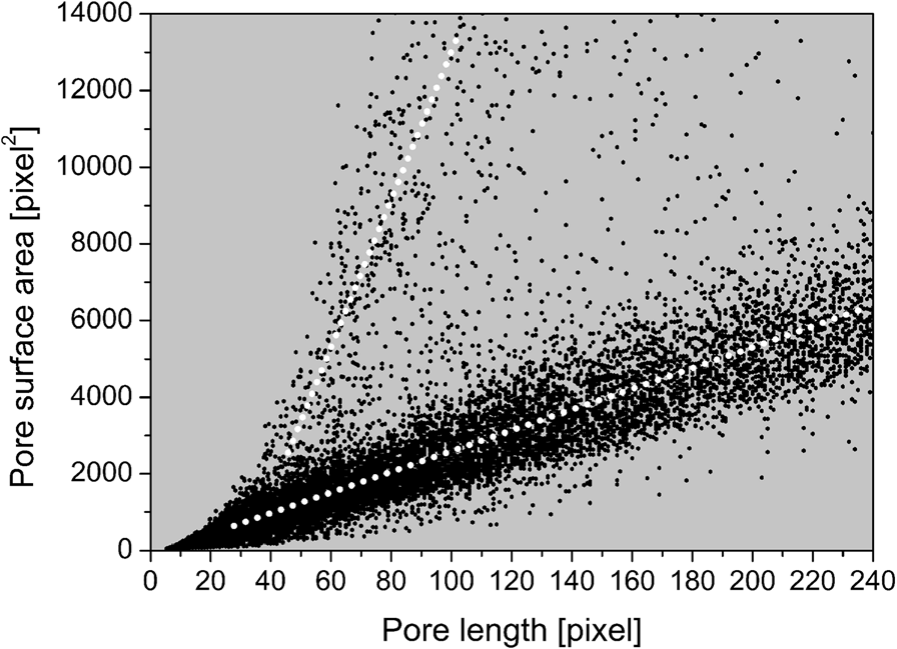
**Relationship between the surface area of individual pores and pore length.** These data represent more than 33,000 pores in the sample.

X-ray tomography combined with imaging processing techniques provides information about each physical feature separately (for example, pore surface area and size), so the relationship between different parameters can be also obtained. Figure 4 shows the linear correlation between the surface area of individual pores and pore length. The data on this dispersion curve are mainly grouped in two regions, along two lines with different slopes; here used only to guide the eyes. For a defined pore length, a small number of internal lumens will have greater surface area than others. This indicates that there is a proportion of pores with physical features that differ considerably from those of the majority present in the piassava fiber. This group of pores is illustrated in Figure 3d. These pores are located in the sclerenchyma and have a shorter aspect ratio than that of the pores in the parenchyma tissue.

**Figure 5:**
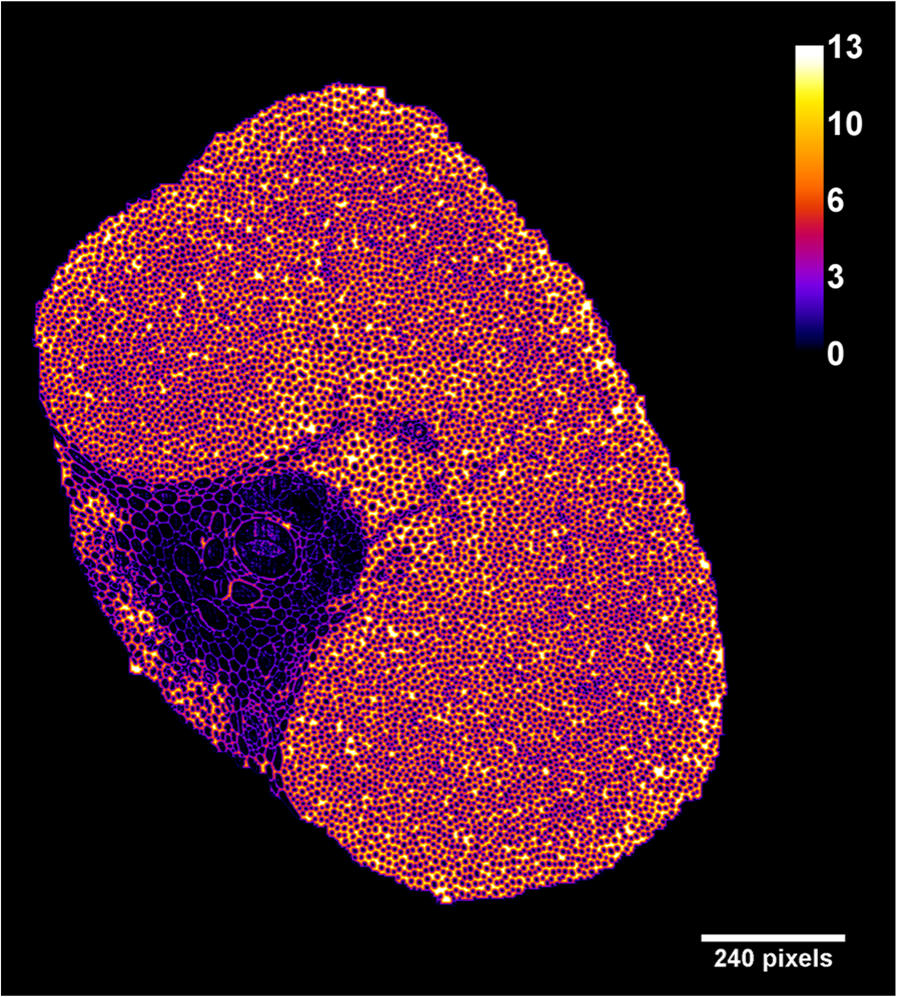
**Map of the distance to the edge of the cell wall of the piassava fiber cross section in pixels.** The legend and scale bar are in pixels (1 pixel = 0.438 μm).

Besides the pore surface area and size, the effect of cell wall thickness on the structural recalcitrance of the lignocellulosic biomass is also discussed [27]. To explore this relationship, a map of the distance to the edge of the cell wall over the fiber cross section was computed (Figure 5). Like other lignocellulosic substrates, the piassava fiber is heterogeneous; the distance to the edge of the cell wall ranges from a few pixels up to 12. The thickness of parenchyma cells is close to 12 pixels (5.25 μm), and cell walls in the vascular bundle and surrounding sclerenchyma are three to four times thinner. It is important to emphasize that these results were obtained for the native structure of the plant cell wall because the sample was not subjected to any preparation process.

Extracting the three-dimensional features of bulk particles can provide extremely valuable information to better understand the substrate and consequently biomass recalcitrance. PCT combined with image processing enabled us to extract individual physical features of the internal lumens of piassava fibers and also to estimate gross characteristics such as the area available to react with enzymes. Many questions such as why it is difficult for some plants, or portions of plants, to release their sugars and what structural features change to render pretreated biomass digestible can be thoroughly investigated using the approach described in this work. By answering these questions, insights to develop more efficient pretreatment processes and less recalcitrant substrates will be provided.

## Conclusions

PCT allowed us to directly visualize the bulk and three-dimensional cell wall architecture in biomass. The imaging and processing techniques used here gave reliable measurements of the surface area of individual pores, pore length, and distance to cell wall edge, which are the parameters required to increase knowledge of structural biomass recalcitrance. In this context, plant cell walls from piassava fibers were used as a model to examine what is currently known about cell wall structures related to biomass.

Three-dimensional imaging revealed the complex and heterogeneous cellular structure of the piassava fibers. Structural features were visualized and the textured external surface could also be resolved because of the extremely high sensitivity of PCT. Statistically relevant information on the surface area, length, and number of pores in bulk biomass was obtained from the tomograms.

Quantitative analyses revealed that the surface area of individual pores in piassava fibers exhibited a bimodal distribution. Structural features (for example, surface area and length of individual pores) could be determined separately, allowing the relationship between different parameters to be determined. A positive linear relationship was found between pore surface area and length.

The total accessible surface area of the piassava fibers directly measured by PCT was of the same order of magnitude as that obtained by the BET method. However, using PCT, the external surface of individual fibers and available surface area of the bulk material at the micrometer scale could be obtained separately. It could be confirmed, therefore, that the external surface of the fiber has only a minor contribution to the gross available surface area.

This investigation is an important step towards understanding the influence of structural and morphological characteristics on biomass recalcitrance. Using PCT, important outstanding questions in the area of recalcitrance and deconstruction of biomass can be clarified, which will provide new insights to obtain more efficient substrates and pretreatment processes. Future experiments will concentrate on strategic feedstocks for ethanol production in large-scale biorefineries such as sugarcane bagasse and corn stover.

## Methods

### Model lignocellulosic biomass

Piassava fibers extracted from the leaves of a palm tree native to the state of Bahia in the northeast region of Brazil were used in this work as model lignocelluloses [17]. The fibers were about 4-m long, with an average diameter of 1.1 mm, and density in the range of 1.10 to 1.45 g/cm^3^ [16]. The fibers possessed a cellulose content of 31.6% and lignin content of 48.4%, which is comparable with that of coir but higher than those of some of the other plant fibers [16].

### Imaging plant cell walls by phase-contrast tomography

The cellular organization in the piassava fibers was studied by PCT using synchrotron radiation. Measurements were performed at the imaging setup of the BAMline at the German storage ring BESSY (Berliner Elektronenspeicherring - Gesellschaft für Synchrotronstrahlung m.b.H.), operated by the HZB (Helmholtz Centre Berlin for Materials and Energy, Berlin, Germany) [28]. During sample rotation, 1,800 radiographic projection images were taken. The X-ray energy used for the measurements was 17 keV. Each voxel represents dimensions of 0.438 × 0.438 × 0.438 μm. Because of the restricted field of view of the CCD camera, piassava fibers with diameters of less than 1 mm were chosen for the tomographic inspections. A phase retrieval algorithm for tomographic reconstruction based on Paganin *et al*. was applied [29].

### Image processing

The evaluation of tomographic volume was focused on the cell wall organization. Voxels were identified as belonging either to pores or plant cell walls by applying an intensity threshold based on their gray level. Information on the number of intercellular spaces (pores) and their geometric properties was extracted from tomograms using a commercial code. The distribution of cell wall thickness was also obtained using ImageJ software.
